# Ribonuclease J is required for chloroplast and embryo development in *Arabidopsis*

**DOI:** 10.1093/jxb/erv010

**Published:** 2015-02-20

**Authors:** Hongyu Chen, Wenxuan Zou, Jie Zhao

**Affiliations:** State Key Laboratory of Hybrid Rice, College of Life Sciences, Wuhan University, Wuhan 430072, China

**Keywords:** *Arabidopsis*, chloroplast, embryo, pattern formation, *RNase J*.

## Abstract

Chloroplasts perform many essential metabolic functions and their proper development is critically important in embryogenesis. However, little is known about how chloroplasts function in embryogenesis and more relevant components need to be characterized. In this study, we show that *Arabidopsis* Ribonuclease J (RNase J) is required for chloroplast and embryo development. Mutation of *AtRNJ* led to albino ovules containing aborted embryos; the morphological development of *rnj* embryos was disturbed after the globular stage. Observation of ultrastructures indicated that these aborted embryos may result from impaired chloroplast development. Furthermore, by analyzing the molecular markers of cell fate decisions (*STM*, *FIL*, *ML1*, *SCR*, and *WOX5*) in *rnj* embryos, we found that this impairment of chloroplast development may lead to aberrant embryo patterning along the apical-basal axis, indicating that AtRNJ is important in initiating and maintaining the organization of shoot apical meristems (SAMs), cotyledons, and hypocotyls. Moreover, the transport and response of auxin in *rnj* embryos was found to be disrupted, suggesting that AtRNJ may be involved in auxin-mediated pathways during embryogenesis. Therefore, we speculate that RNJ plays a vital role in embryo morphogenesis and apical-basal pattern formation by regulating chloroplast development.

## Highlight

*Arabidopsis* RNase J plays an important role in maintaining chloroplast function, and the impairment of chloroplast development in *rnj* mutants may lead to aberrant embryos with disrupted pattern formation.

## Introduction

In higher plants, embryogenesis starts from a single fertilized egg cell (zygote). *Arabidopsis* zygotes undergo a series of programmed cell divisions and differentiation processes that result in the formation of mature embryos. Although some of the molecular factors that regulate embryo patterning have been identified (Capron *et al.*, 2009; Jeong *et al.*, 2011; Wendrich and Weijers, 2013; Costa *et al.*, 2014; Huang *et al.*, 2014), the overall process and particular mechanisms involved remain poorly understood. Since the release of the *Arabidopsis* genome sequence in 2000 and the isolation and analysis of the *embryo-defective* (*emb*) mutants, a large number of genes involved in embryogenesis have been characterized as essential. Many such genes were predicted through enrichment analysis to function in basic cellular processes such as DNA, RNA, and protein synthesis, and these genes are likely to have counterparts in yeast and *Caenorhabditis elegans*. Fewer regulatory genes, such as transcription factors and signalling components, were identified in this analysis (Tzafrir *et al.*, 2004). It has been suggested that embryogenesis is precisely controlled by a complicated network of gene expression, and that about 1000 genes play crucial roles during *Arabidopsis* embryo development (Meinke *et al.*, 2008). Only a portion of these genes have been studied experimentally in detail. The characterization and functional dissection of ever more unknown yet essential genes will deepen our understanding of the molecular mechanisms and regulatory networks underlying embryogenesis.

Many of the essential genes have been shown to encode proteins that are localized in chloroplasts (Bryant *et al.*, 2011). During *Arabidopsis* embryogenesis, chloroplasts are derived from the proplastids in meristematic cells when the embryo develops up to the late globular stage (Mansfield and Briarty, 1991). The proplastid is a kind of undifferentiated plastid with very few internal membrane vesicles; it is small and colourless. In response to light, the invaginated inner envelope of proplastids enfolds to form more vesicles that are linked and merged together, finally resulting in the development of thylakoid membranes (López-Juez, 2007). Accompanied by the accumulation of proteins and lipids required for photosynthesis, chlorophyll starts to be synthesized in heart stage embryos; subsequent photosynthesis in chloroplasts supplies energy for later stages of embryo development. The synthesis of compounds that are critical for embryogenesis, including amino acids, lipids, and phytohormones, also occurs in chloroplasts (Ruuska *et al.*, 2004).

A comprehensive data set of 119 nuclear genes encoding *Arabidopsis* chloroplast proteins associated with embryo development was established by querying the literature and the SeedGenes database (Tzafrir *et al.*, 2004; Bryant *et al.*, 2011). These genes/proteins were then divided into three major groups according to their probable functions: (i) enzymes required for the biosynthesis of amino acids, vitamins, nucleotides, and fatty acids; (ii) essential proteins required for the import, modification, and localization of chloroplast proteins; and (iii) proteins required for expression of the chloroplast genes. It is known that there are many diverse chloroplast proteins that are required for embryo development in *Arabidopsis*. The elimination of biosynthetic functions within the chloroplast and interferance with the expression of chloroplast genes often results in embryo death. Several chloroplast proteins that are known to perform crucial functions in embryogenesis have recently been studied; examples include Hsp90C (Inoue *et al.*, 2013), PGDH1 (Benstein *et al.*, 2013), and NAD–MDH (Selinski *et al.*, 2014). An essential role for *Arabidopsis* chloroplast heat shock protein 90 (Hsp90C) in protein import into chloroplasts had been demonstrated; the siliques from heterozygous *hsp90c* plants contained ~25% albino and aborted seeds that failed to develop properly. *Arabidopsis* phosphoglycerate dehydrogenase1 (PGDH1) is localized in plastids and required for ammonium assimilation and tryptophan biosynthesis. Analysis of *pgdh1* mutants revealed an embryo-lethal phenotype. The plastid-localized NAD-dependent malate dehydrogenase (NAD–MDH) enzyme is crucial for energy homeostasis in developing *Arabidopsis* seeds. Embryos in homozygous knockout seeds only grew to reach the globular stage and the seeds developed into tiny wrinkled shapes. These results demonstrate that chloroplast proteins play essential roles in embryo development.

Chloroplasts originated when a photosynthetic prokaryote was engulfed and ‘enslaved’ by the primitive eukaryotic ancestor of land plants. They are semiautonomous organelles and contain their own genomes. Most of the endosymbiotic prokaryote genes were transferred to the nuclear genome or lost, though modern chloroplasts retain some metabolic activities, genetic mechanisms, and protein transport complexes that clearly reflect their prokaryotic origins (Reyes-Prieto *et al.*, 2007; Keeling, 2010). RNase J is a well studied nuclease that plays an important role in RNA metabolism in bacteria (Mathy *et al.*, 2007). In *Arabidopsis*, this protein has exoribonuclease activity and compensates for inefficient transcription termination by removal of antisense RNA in chloroplasts. Therefore, it plays an important role in the regulation of chloroplast gene expression (Sharwood *et al.*, 2011). However, the biological function of AtRNJ in embryogenesis is still unknown.

In this study, we found that plants are the only eukaryotes that have genes encoding RNJs. Further, we observed that RNJ family members from both monocots and eudicots all display a high degree of sequence homology. *AtRNJ* is highly expressed in green tissues and reproductive organs, and its expression level is greatly dependent on light. Three null *rnj* mutants all displayed the same phenotype: about 25% aborted seeds distributed in siliques of heterozygous plants, and no homozygous plants could be identified. Furthermore, we show that chloroplast development in *rnj* embryo cells is impaired. Mutation of the *RNJ* gene caused impediments in cell differentiation, apical-basal patterning, and auxin metabolism during early embryogenesis. Therefore, AtRNJ plays a vital role in chloroplast development and in embryo cell fate determination.

## Materials and methods

### RNJ sequence analysis

We obtained the protein sequences of the *Arabidopsis RNJ*, *CPSF73-I*, *CPSF73-II*, and *TRZ* genes from the Arabidopsis Information Resource (http://www.arabidopsis.org), and the sequences of their homologues in various species from the National Center for Biotechnology Information by data bank searching using BLAST (http://blast.ncbi.nlm.nih.gov/Blast.cgi). Multiple sequence alignment was performed on ClustalX (version 1.83), and MEGA4 software was used to display the phylogenetic tree based on the Neighbour-Joining method (Tamura *et al.*, 2007).

### Plant materials and growth conditions

The three *Arabidopsis thaliana* L. *rnj* alleles, *rnj-1* (CS16191), *rnj-2* (CS815990), and *rnj-3* (CS24091), were obtained from the Arabidopsis Biological Resource Center (http://abrc.osu.edu/). The T-DNA insertion sites in the three mutants were confirmed by PCR and sequencing. Plant lines carrying *pDR5rev::3XVENUS-N7*, *pPIN1::PIN1-GFP*, *pSTM::STM-VENUS*, and *pFIL::dsRED-N7* (Heisler *et al.*, 2005) were obtained from Elliot Meyerowitz (Division of Biology, California Institute of Technology, CA, USA); the *pSCR::H2B-YFP* and *pWOX5::GFP* lines (Heidstra *et al.*, 2004; Blilou *et al.*, 2005) were obtained from Ben Scheres (Department of Biology, University of Utrecht, The Netherlands); and the *pAtML1::NLS-3xEGFP* line (Takada and Jürgens, 2007) was obtained from Gerd Jürgens (Developmental Genetics, Centre for Molecular Biology of Plants, University of Tübingen, D-72076 Tübingen, Germany). The *Arabidopsis* plants were cultivated in a greenhouse at Wuhan University at 22±2°C with a 16-h light/8-h dark cycle.

Plant lines carrying different markers were crossed with *rnj-2* heterozygotes, and the progenies were identified by PCR and observed under a fluorescence microscope. The *rnj-2*/+ mutants carrying a homozygous fluorescence marker were used for subsequent experiments.

For dark treatment, *Arabidopsis* seeds were sterilized and plated on solid 1/2 MS medium. After stratification for 2 days at 4°C, the seeds grew under the normal light conditions for 6 days at 22°C. At 6 days after germination (DAG), seedlings were transferred to dark conditions for 1 day, while the control-tested seedlings grew under the normal light cycle.

### RT-PCR and quantitative RT-PCR

Total RNA from different kinds of *Arabidopsis* tissue were isolated using TRIZOL reagent (Sigma, http://www.sigmaaldrich.com/), and transcribed into cDNA with a Reverse Transcription System (TOYOBO, http://www.toyobo.co.jp/e/) after digestion with DNase I (Fermentas). Next, the cDNA was used as the template for PCR analysis with gene-specific primers (Supplementary Table S1). Real-time PCR was performed using TransStart Top Green qPCR SuperMix (TransGen, China) with a Rotor-Gene 6000 machine (Corbett Research, http://www.corbettlifescience.com/). At least two independent biological replicates were made for quantitative PCR analysis, and three technical replicates were taken in each biological replicate. The *GAPC* was applied as a reference gene for quantitative PCR analysis. The relative expression levels were analysed as described by Ren *et al.* (2012).

### *RNJ* genomic DNA cloning and *rnj* mutant complementation

An *RNJ* genomic DNA fragment including 2100bp of the promoter and 5′-UTR region was cloned from wild-type genomic DNA using a pair of specific primers (Supplementary Table S1). The amplified DNA fragment was verified by sequencing, cloned into pCambia1300 vector (Cambia, http://www.cambia.org/), and transferred into three *rnj*/+ mutants by the floral-dip method (Clough and Bent, 1998). After screening the transgenic seeds on hygromycin plates, positive transformants were identified by PCR, and then used for subsequent analysis.

### Ovule clearing and embryo observation

Fresh ovules were dissected from siliques using forceps and mounted in Hoyer’s solution [chloral hydrate:glycerol:water, 8:1:2 (w/v/v)] for minutes to hours depending on the embryo developmental stage (Berleth and Jurgens, 1993). Next, the cleared ovules with embryos were examined by differential interference contrast microscopy under an inverted microscope (Olympus TH4-200; http://www.olympus-global.com/) equipped with a CCD of SPOT Digital Microscope Camera (Diagnostic Instruments, http://www.spotimaging.com/).

### RNJ promoter and GUS/GFP fusion

The promoter fragment of *RNJ* was amplified with genome-specific primers (Supplementary Table S1). After verification by sequencing, the amplified DNA fragment was cloned into pCAMBIA1381Xb and pCBIm–eGFPm binary vector (Cambia, http://www.cambia.org/), and then transformed into *Arabidopsis* plants using the method described above.

### GUS staining analysis

The homozygous T4 generation *pRNJ::GUS* transformants were used for GUS staining analysis. The GUS staining was carried out as described by Yuan *et al.* (2008). The samples were observed under an Olympus SZX12 stereomicroscope (http://www.olympus-global.com/en/) and photographed using a digital camera (Cool SNAP, RS Photometric; http://www.photometrics.com/products/ccdcams).

Confocal laser scanning microscopyConfocal laser scanning microscopy (CLSM) was used to detect the fluorescent signal of molecular markers and transgenic lines. Fresh embryos were isolated from ovules, mounted in 10% glycerol, and then observed under an Olympus FV1000 confocal microscope.

### Transmission electron microscopy

The 5 DAP ovules of wild-type and *rnj* mutants were fixed, embedded, and sectioned as described by Qin and Zhao (2006), and then the prepared samples were observed and photographed using transmission electron microscopy (TEM; TEHitachi H-7000 FA).

## Results

### RNase J is a metallo-beta-lactamase that is conserved in plants

**Fig. 1.**
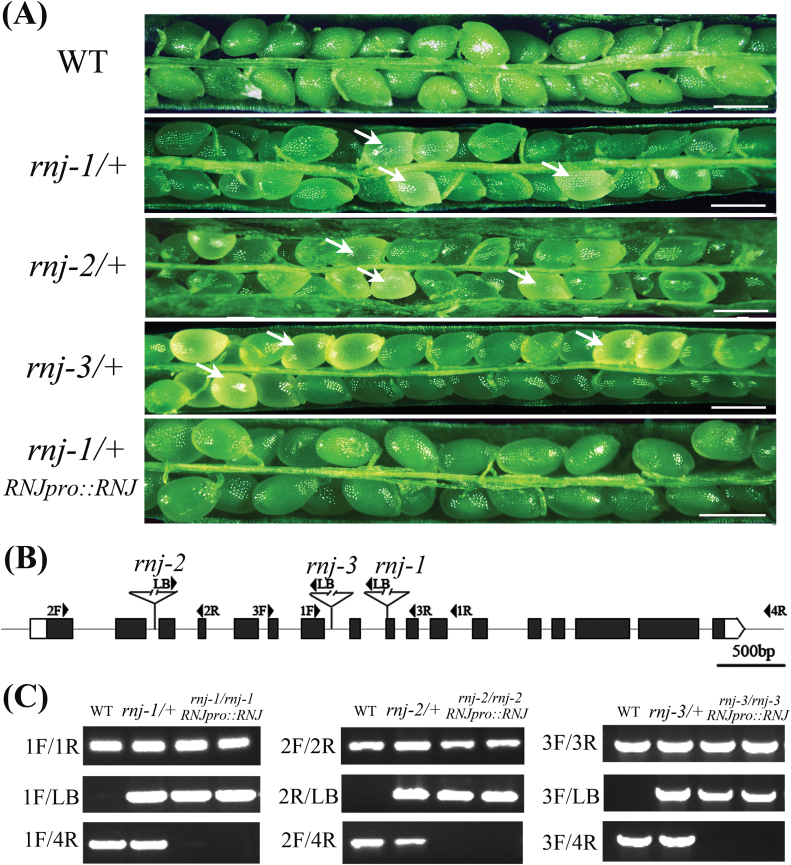
T-DNA insertion mutants of *RNJ* and functional complementation. (A) Seed development in wild-type, *rnj-1*/+, *rnj-2*/+, *rnj-3*/+, and functionally complemented *rnj-1*/+ transgenic plants. The arrows show the aborted white ovules. Scale bars = 0.5mm. (B) Schematic diagram of three T-DNA insertions in the *Arabidopsis RNJ* gene. The three mutants *rnj-1* (CS16191), *rnj-2* (CS815990), and *rnj-3* (CS24091) have T-DNA insertions in exon 9, intron 2, and intron 7, respectively. Black boxes indicate exons, grey lines indicate introns, and arrowheads indicate the positions of primers used for genotyping. (C) PCR analysis of three *rnj* mutants and functionally complemented transgenic plants. This figure is available in colour at *JXB* online.

RNase J (RNJ) is a member of the metallo-beta-lactamase protein family that has been studied extensively in bacteria (Bebrone, 2007). It plays an important role in rRNA maturation and in the 5′ stability of mRNA (Mathy *et al.*, 2007). Cloning of the *Arabidopsis RNJ* gene (At5g63420) from genomic DNA and cDNA confirmed that it contains 17 exons and 16 introns (Fig. 1B). Bioinformatic analysis showed that there are three other types of RNJ-like metallo-beta-lactamases involved in RNA metabolism in eukaryotes: TRZ (tRNase Z), CPSF73-I (cleavage and polyadenylation specificity factor 73-I), and Int11 (Integrator 11). The TRZ and CPSF73-I proteins can be found in plants, humans, and yeast, while Int11 can be found in plants and humans but not in yeast; this protein was first studied and designated as CPSF73-II in *Arabidopsis* (Supplementary Figure 1A). However, the RNJs are only encoded in plants among eukaryotes, and AtRNJ displays a high degree of homology with other RNJs in both monocots and eudicots (Supplementary Figure 1A). The fact that no proteins homologous to RNJ are found in animal or yeast genomes suggests that this protein may play a special function in plants. Protein comparison showed the five signature motifs as described by Dominski (2007), indicating that the four tRNase Z proteins are metallo-beta-lactamase (Supplementary Figure 1B). The most characteristic feature of the RNJ, CPSF73-I, and CPSF73-II proteins is the lack of a readily identifiable motif 5 and instead the presence of three conserved motifs, A–C (Supplementary Figure 1B).

### Homozygous *rnj* mutation causes seed abortion

To investigate the biological functions of the *Arabidopsis RNJ* gene, we obtained three independent T-DNA insertion mutants from the Arabidopsis Biological Resource Center (http://abrc.osu.edu/). The positions of the T-DNA insertions in *rnj-1* (CS16191), *rnj-2* (CS815990), and *rnj-3* (CS24091) mutants were verified by genomic PCR and sequencing; these insertions where found to be located in exon9, intron2, and intron7, respectively (Fig. 1B). The functional domains of RNJ with respect to the T-DNA insertions are also indicated in Supplementary Figure 2. Genotypic analysis of the *rnj*/+ progeny (*n* > 100 per line) showed no homozygotes for any of the three mutants. None of the heterozygous plants exhibited any vegetative developmental defects, but a portion of the ovules in mature siliques were white (Fig. 1A), with frequencies of 24.9% in *rnj-1*/+ (*n* = 1653, *P* = 0.99), 25.7% in *rnj-2*/+ (*n* = 2422, *P* = 0.48), and 25.1% in *rnj-3*/+ (*n* = 1372, *P* = 0.96). The white ovules in *rnj*/+ siliques then turned brown and wrinkled, occurring at a frequency close to the expected value of 25% (Tzafrir *et al.*, 2004). This finding suggests that the homozygous *rnj* mutation causes seed abortion.

To clarify whether a null mutation in *RNJ* could result in gametophyte sterility, we performed further genetic analysis on the *rnj-2*/+ lines. The T-DNA insertion in *rnj-2*/+ harbours a genetic tag of Basta (Bas) resistance for the mutant plants; this feature facilitates segregation analysis of mutant alleles. The progeny seedlings of self-pollinated *rnj-2*/+ plants segregated close to a 2:1 ratio of Bas resistant (Bas^R^) to Bas sensitive (Bas^S^) (Table 1). Because no homozygous seedlings were obtained, this segregation ratio was taken as being indicative of the expected theoretical ratio of 2:1 for heterozygous to wild-type plants. To determine whether T-DNA could be transmitted through the male and female gametophytes, we performed reciprocal crosses with *rnj-2*/+ and wild-type plants (Meinke *et al.*, 2008). When *rnj-2*/+ plants were used as recipients in crosses with wild-type pollen, 48.3% of progeny were resistant to Basta and the transmission efficiency of female gametophytes was 93.5% (Table 1). When wild-type pistils were crossed with the *rnj-2*/+ pollen grains, 52.3% of progeny were resistant to Basta and the transmission efficiency of male gametophytes was 109.5% (Table 1). These results indicate that the transmission efficiency of the *rnj-2*/+ mutation was normal through both male and female gametophytes.

**Table 1.** Transmission analysis of reciprocal crosses between *Arabidopsis* wild type and *rnj-2*/+

Female × Male	BASTA^R^	BASTA^S^	BASTA^R^:Total	TE (%)	*P* value
rnj-2/+ × rnj-2/+	1249	628	0.67:1	NA	NA
rnj-2/+ × +/+	288	308	0.48:1	93.5	0.24
+/+ × rnj-2/+	243	222	0.52:1	109.5	0.18

BASTA^R^, BASTA resistant; BASTA^S^, BASTA sensitive; NA, not applicable; TE, transmission efficiency [(BASTA^R^/BASTA^S^) × 100]. The *P* value is based on an expected 100% TE; *P* > 0.05 indicates no significant differences.

To confirm that the observed seed lethality was caused by interruption of the *RNJ* gene, we employed a complementation strategy to test whether or not the full-length genomic sequence of *RNJ* could rescue the seed-lethal phenotype. The genomic fragment of *RNJ*, including the 5772bp gene sequence and 2100bp upstream of the ATG codon, was introduced into each of the three mutants (*rnj-1*/+, *rnj-2*/+, *rnj-3*/+). PCR screening and phenotypic analysis in the T2 progeny of the complementation lines allowed us to identify homozygous *rnj* mutants that showed no aborted seeds in siliques (Fig. 1A, C). These results indicated that *RNJ* was indeed the gene responsible for the seed lethality and that it is an essential gene in seed development.

### Morphological development of the *rnj* mutant embryo is disturbed

**Fig. 2.**
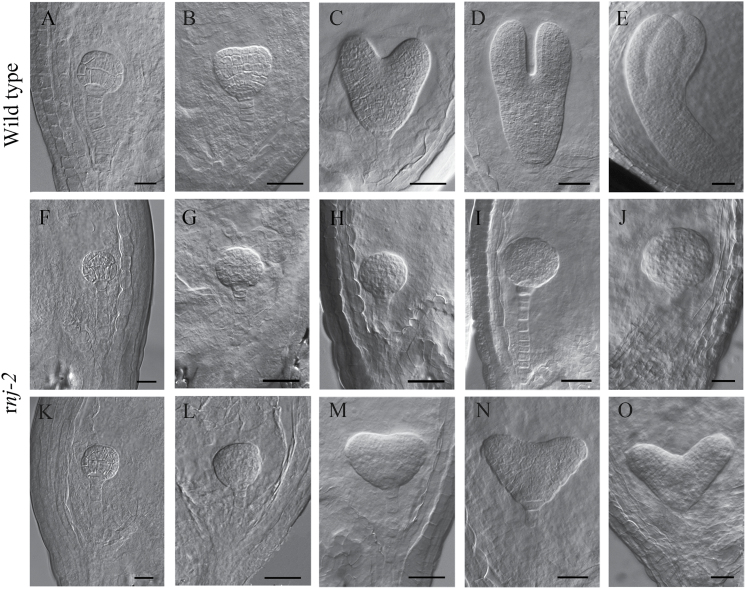
Embryogenesis in wild-type and *rnj-2*/+ plants examined by differential interference contrast microscopy. (A–E) Embryos from the globular stage to the bent cotyledon stage in wild-type ovules: (A) globular stage; (B) transition stage; (C) heart stage; (D) torpedo stage; (E) bent cotyledon stage. (F–J) The irregular globular *rnj* embryos from the same siliques at the different development stages as in (A–E). (K–O) The *rnj* embryos with abnormal cotyledons from the same siliques at the different development stages as in (A–E). Scale bars = 20 μm.

To investigate how the seed abortion phenotype occurred in the siliques of *rnj* heterozygotes, we examined the seed developmental processes in wild-type and *rnj-2*/+ plants with a whole mount clearing technique and differential interference contrast microscopy. The results showed that there were no obvious differences between wild-type and *rnj-2* mutant embryos from the zygote up to the early globular stage (Fig. 2A, F, and K). Subsequently, disturbed embryos were observed in the three mutants; aberrant phenotypes became more obvious as embryo development progressed. By the time that the wild-type embryos had developed to the heart stage, two kinds of aborted embryos were distinguishable: irregular globular embryos and abnormal cotyledon embryos. The irregular globular embryos showed shape alterations and abnormal cell division and were unable to produce cotyledons (Fig. 2G–J). The abnormal cotyledon embryos had two cotyledons with unequal and asymmetric growth, accompanied by a much larger angle between the two cotyledons, as compared to wild-type embryos (Fig. 2L–O). Of 244 mutant embryos at 5 DAP, 185 (75.8%) embryos were irregularly globular, while 59 (24.2%) had abnormal cotyledons. The same embryonic defects could also be observed in *rnj-1* and *rnj-3* mutants (Supplementary Figure 3), suggesting the *RNJ* gene function is totally lost in each of the three alleles. These results showed that the homozygous *rnj* mutation caused embryo abortion at the late globular stage and that many *rnj* mutant embryos could not develop beyond this stage, indicating that morphological development of the embryos was disturbed in the *rnj* mutants.

### *RNJ* is expressed in green tissues and reproductive organs

**Fig. 3.**
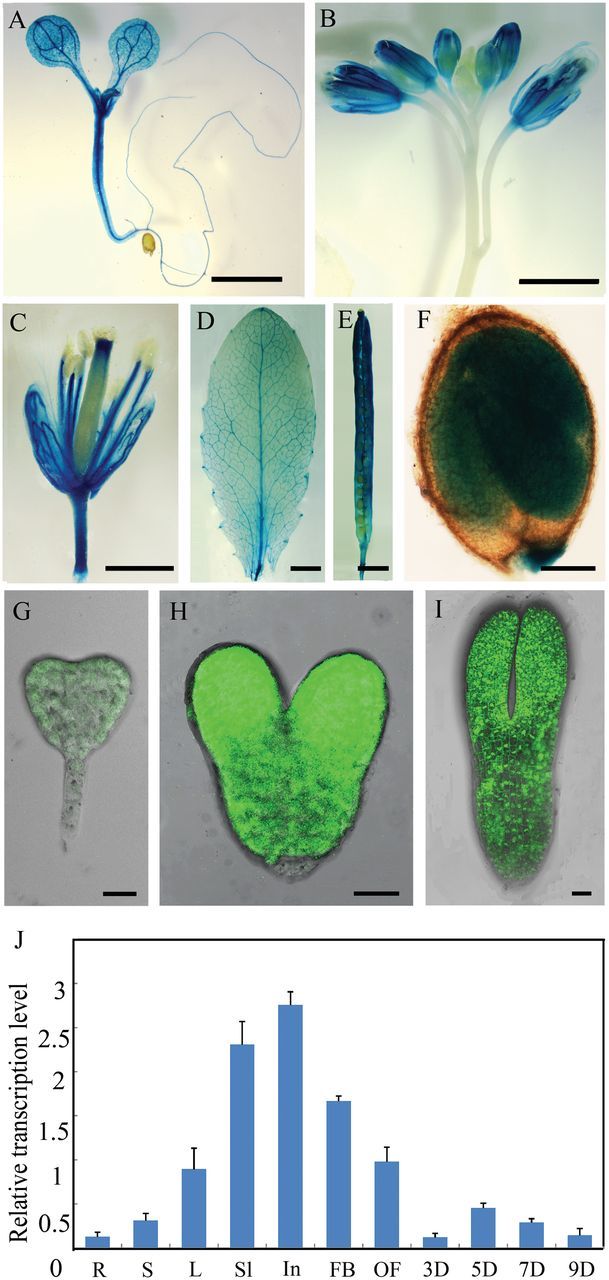
Expression pattern analysis of *RNJ* in *Arabidopsis*. (A–F) GUS staining signals in *pRNJ::GUS* transgenic plants: (A) 7 DAG seedling; (B) inflorescence; (C) flower; (D) rosette leaf; (E) adult silique; (F) mature seed. (G–I) GFP images of embryos from *pRNJ::GFP* transgenic plants: (G) transition stage; (H) heart stage; (I) torpedo stage. (J) Quantitative PCR analysis of *RNJ* in various tissues. R, root; S, stem; L, leaf; Sl, seedling; In, inflorescence; FB, flower bud; F, flower; 3Si, 3 DAP silique; 5Si, 5 DAP silique; 7Si, 7 DAP silique; 9Si, 9 DAP silique. Scale bars (A–E) = 2mm; scale bars (F–I) = 20 μm.

To analyse the expression pattern of the *RNJ* gene, we used quantitative PCR to evaluate its mRNA levels in different tissues and organs, with *GAPC* used as a reference gene. The results showed that *RNJ* was expressed at different levels in nearly all organs, and the relative expression levels were most abundant in inflorescences and seedlings (Fig. 3J). To investigate the spatial expression pattern, we fused the *RNJ* promoter (2100bp) with a *β*-glucuronidase (GUS) reporter gene to monitor its expression in transgenic plants (*pRNJ::GUS*). In 7-day-old seedlings, strong GUS signals were detected in shoot meristems, hypocotyls, and in the vascular bundles of cotyledons, as well as in the veins of mature leaves (Fig. 3A, D). In reproductive organs, GUS expression was detected in inflorescences, especially in sepals, filaments, and stigmas (Fig. 3B, C), as well as in mature siliques and seeds (Fig. 3E, F). We also fused the *RNJ* promoter with green fluorescent protein (GFP) to evaluate the expression of *RNJ* in more detail during embryo development. During the early stages of embryo development, no GFP signal could be detected until the transition stage (Fig. 3G). In the heart and torpedo stages, GFP fluorescence was predominantly distributed in the upper part of embryos (Fig. 3H, I). These results showed that *RNJ* is expressed widely in green tissues and reproductive organs, and that it is expressed at particularly high levels in the heart and torpedo embryos as compared to the transition stages.

A previous study confirmed that the *Arabidopsis* RNJ protein is localized in the chloroplast (Sharwood *et al.*, 2011). In this study, we found 18 light-response elements in the *RNJ* promoter sequence (http://arabidopsis.med.ohio-state.edu/AtcisDB/), including the GATA factors that were reported by Teakle *et al.* (2002). We therefore wondered whether or not *RNJ* expression was influenced by light. Quantitative PCR was performed to detect the mRNA levels in the 7 DAG seedlings. After 1 day of dark treatment, the expression level of the *RNJ* gene decreased by 67.3% compared to the controls (Supplementary Figure 4A). GUS staining also indicated obviously lower expression levels in dark-treated *pRNJ::GUS* transgenic seedlings than in the controls (Supplementary Figure 4B, C). These results verified our supposition that light is an important signal in the regulation of *RNJ* expression. Since the GATA factors are type-IV zinc-finger proteins with DNA-binding and transcriptional activation activities, and several GATA factor genes had been verified to be in response to light (Manfield *et al.*, 2007), we suggest that *RNJ* expression may be under the indirect control of light, perhaps via the products of photosynthesis.

### Chloroplast development is impaired in homozygous *rnj* embryos

**Fig. 4.**
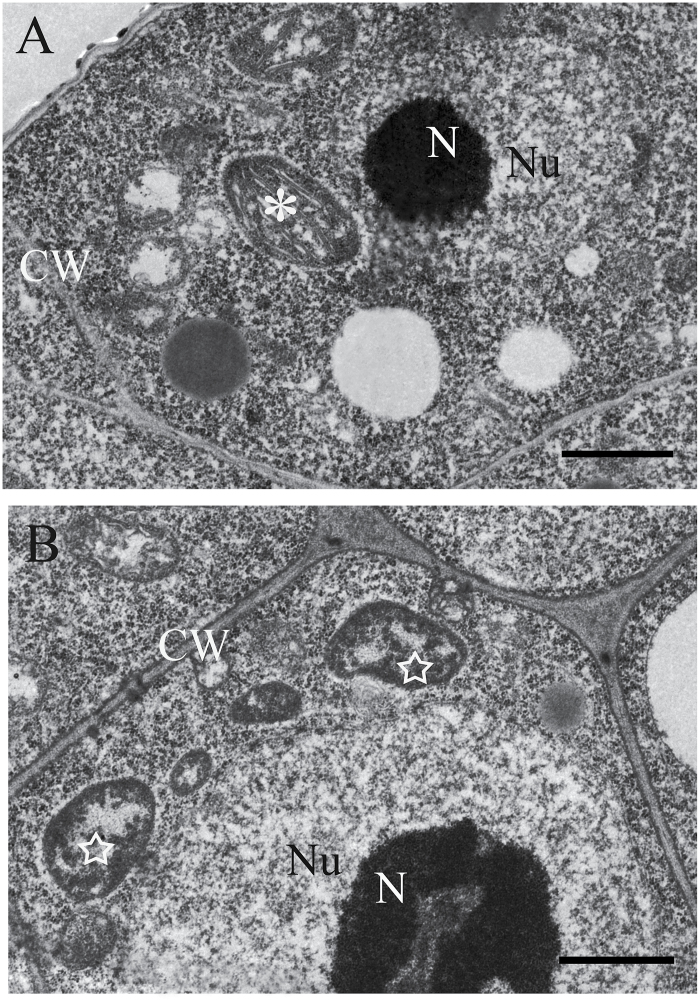
TEM of chloroplast development in wild-type and *rnj-2* embryo cells. (A) Cells in wild-type embryos at 5 DAP. (B) Cells in *rnj-2* embryos at 5 DAP. Asterisk indicates a normal chloroplast in the wild type; star indicates an impaired chloroplast in the *rnj-2* mutant. N, nucleolus; Nu, nucleus; CW, cell wall. Scale bars = 1 μm.

Since the *rnj* heterozygous mutants produced about 25% albino seeds in siliques, we wondered whether the homozygous embryo lethality was due to impaired chloroplast development in the mutant embryo cells. We prepared wild-type and *rnj* albino ovules from 5 DAP siliques as samples for ultrastructural observation with TEM. In the embryo wild-type samples, chloroplasts had organized thylakoid membranes stacked into grana that were well developed (Fig. 4A). However, in the *rnj* embryo samples, we observed many immature plastids that lacked internal thylakoid membranes but contained darkly stained aggregations (Fig. 4B). The lack of normal chloroplasts in the mutant embryo cells indicated that the *rnj* mutation may disturb the formation of internal thylakoid membranes during embryo development and lead to impaired chloroplasts, strongly suggesting that the *RNJ* gene is required for chloroplast development.

### The apical-basal patterning of the *rnj* embryo is perturbed

The phenotypes observed in *rnj* embryos showed a morphologically defective transition from globular to bilateral symmetry along the apical-basal axis; there were obvious problems with the specification of shoot apical meristem (SAM), cotyledon, and hypocotyl development. To better understand the patterning defects in *rnj* embryos, we investigated the expression patterns of several genes known to delineate fate decisions in the embryo by using fluorescent markers.

**Fig. 5.**
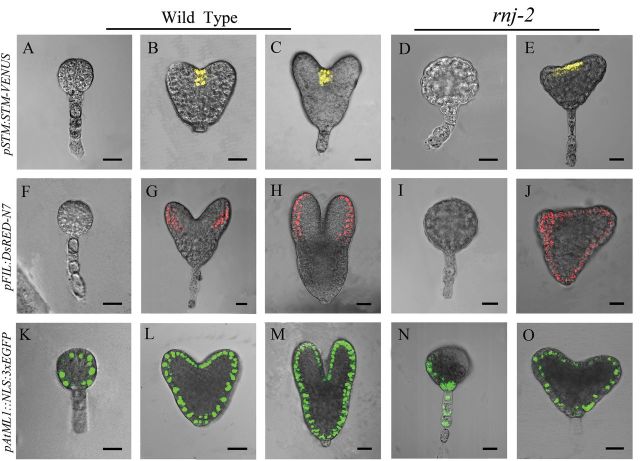
Expression pattern of *STM*, *FIL*, and *ML1* genes in wild-type and *rnj* embryos. (A–C) *STM* expression in wide-type embryos of globular stage (A), heart stage (B), and torpedo stage (C). (D, E) *STM* expression in *rnj-2* irregular globular (D) and abnormal cotyledon (E) embryos. (F–H) *FIL* expression in wild-type embryos of globular stage (F), heart stage (G), and torpedo stage (H). (I, J) *FIL* expression in *rnj-2* irregular globular (I) and abnormal cotyledon (J) embryos. (K–M) *ML1* expression in wild-type embryos of globular stage (K), heart stage (L), and torpedo stage (M). (N, O) *ML1* expression in *rnj-2* irregular globular (N) and abnormal cotyledon (O) embryos. Scale bars = 20 μm.

*SHOOT MERISTEMLESS* (*STM*) is a well studied gene expressed in shoot meristems that is required for meristem function (Long and Barton, 1998). Translational fusion of *STM* (*pSTM::STM-VENUS*) to the YFP variant VENUS (Heisler *et al.*, 2005) was used to examine its expression in *rnj* embryos. In wild-type and *rnj* globular embryos, no *pSTM::STM-VENUS* signal could be detected (Fig. 5A, D). After the transition stage, the signal was observed in the central apical region of wild-type embryos (Fig. 5B, C). However, *STM* expression appeared in only one or two layers of cells in the expanded shoot meristems of *rnj* embryos with abnormal cotyledons (Fig. 5E). These results suggested that mutation of the *RNJ* gene perturbs the expression domain of the SAM-organizing gene *STM*.

Since the *rnj* mutant embryos appeared to have disordered or even no cotyledons, we examined the expression of the YABBY gene *FILAMENTOUS FLOWER* (*FIL*), which is known to be specifically expressed on the abaxial side of embryo cotyledon primordia (Siegfried *et al.*, 1999), to determine whether this phenotype was due to defects in cotyledon organogenesis. In wild-type embryos, expression of *FIL* (*pFIL::dsRED-N7*) was restricted to two peripheral domains of developing cotyledons in the apical region after the transition stage (Fig. 5G, H). There was no red fluorescence in wild-type globular embryos (Fig. 5F) or in the irregular globular embryos of the *rnj* mutants (Fig. 5I). Rather, red fluorescence was ectopically distributed in outer cell layers of the embryos with abnormal cotyledons (Fig. 5J). This result suggested that disordered development of cotyledons may be associated with changes in *FIL* expression patterns.

*AtML1* encodes an HD-ZIP-type homeodomain protein. *AtML1* transcript expression is restricted to the outermost or epidermal cell layer embryos at different developmental stages. It is known to regulate epidermal cell fate determination of embryos in *Arabidopsis* (Takada and Jürgens, 2007). In wild-type plants in our experiments, *AtML1* expression (*pAtML1::NLS-3xEGFP*) was detected specifically in the outermost cell layer of embryos from the early globular stages onwards (Fig. 5K–M). *AtML1* expression was confined to the epidermal cell layer in the *rnj* embryos with abnormal cotyledons (Fig. 5O). However, the fluorescent signal only appeared in part of the outermost cells in most of the irregular globular *rnj* embryos (Fig. 5N), suggesting that cell differentiation in the outermost layer may be defective.

**Fig. 6.**
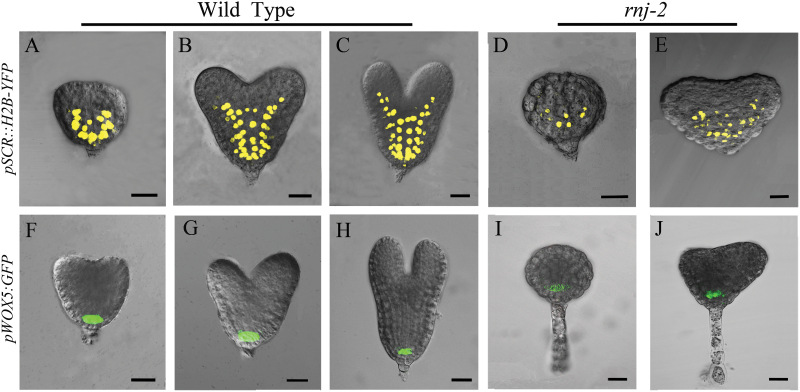
Expression pattern of *SCR* and *WOX5* in wild-type and *rnj* embryos. (A–C) *SCR* expression in wild-type embryos of transition stage (A), heart stage (B), and torpedo stage (C). (D, E) *SCR* expression in *rnj-2* irregular globular (D) and abnormal cotyledon (E) embryos. (F–H) *WOX5* expression in wild-type embryos of transition stage (F), heart stage (G), and torpedo stage (H). (I, J) *WOX5* expression in *rnj-2* irregular globular (I) and abnormal cotyledon (J) embryos. Scale bars = 20 μm.

Since the apical patterning in *rnj* embryos was perturbed, we checked the expression of *SCARECROW* (*SCR*) and *WUSCHEL-LIKE HOMEOBOX5* (*WOX5*) to determine whether the pattern formation in central and basal regions was altered. The *SCR* gene encodes a GRAS transcription factor that is only expressed in the endodermal cell layer (Wysocka-Diller *et al.*, 2000). A transcriptional fusion of SCR to YFP (*pSCR::H2B-YFP*) (Heidstra *et al.*, 2004) was introduced into *rnj* mutants to examine the *SCR* expression pattern. The results showed that *SCR* signals could be detected in the endodermal cells located at the central and basal domains of wild-type and *rnj* embryos (Fig. 6A-E). However, the fluorescent signal in *rnj* embryos showed abnormal cell division (Fig. 6D, E), which led to a decreased number of endodermal cells as well as a shortened embryo. These results suggested that pattern formation in the central region of the *rnj* embryos was disrupted.

We also examined the expression pattern of the *WOX5* gene, a homeodomain transcription factor, in embryos of the wild type and *rnj* mutant using a transcriptional fusion of the *WOX5* promoter in front of GFP (*pWOX5::GFP*) (Blilou *et al.*, 2005). *WOX5* is expressed specifically in the quiescent centre (QC) and is known to be involved in the maintenance of root stem cells (Haecker *et al.*, 2004; Sarkar *et al.*, 2007). Our results showed that the expression domain of *pWOX5::GFP* was not altered and was confined to the QC in both the *rnj* and wild-type embryos (Fig. 6F–J). Our study of *SCR* and *WOX5* expression in *rnj* mutants indicated that formation of the endodermis in the central region of *rnj* embryos was altered, but that there was no alteration of the QC in the basal region in mutant embryos, suggesting that *RNJ* gene expression is required for hypocotyl formation in the central domain but is not necessary for establishment of the embryonic root.

### The *rnj* mutation disrupts auxin transport and responses during embryo development

**Fig. 7.**
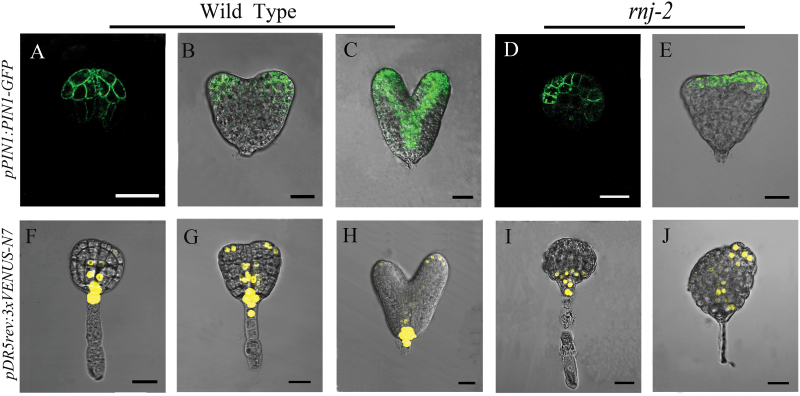
Auxin transportation and distribution is disrupted during embryogenesis in *rnj* mutants. (A–C) *PIN1* expression in wild-type embryos of globular stage (A), transition stage (B) and heart stage (C). (D–E) *PIN1* expression in *rnj-2* irregular globular (D) and abnormal cotyledon (E) embryos. (F–H) *DR5* expression in wild-type embryos of globular stage (F), transition stage (G), and heart stage (H). (I–J) *DR5* expression in *rnj-2* irregular globular (I) and abnormal cotyledon (J) embryos. Scale bars = 20 μm.

Auxin is a key regulator in the control of bilateral symmetry and the establishment of embryo patterning during embryogenesis (Liu *et al.*, 1993). To test whether the developmental defects observed in *rnj* embryos were associated with changes in auxin transport and responses, we analysed *pPIN1::PIN1-GFP* localization and *pDR5rev::3XVENUS-N7* expression in *rnj* embryos. PIN1 is an important auxin efflux transporter that mediates the establishment of the auxin maxima by polar localization (Paponov *et al.*, 2005). In wild-type globular embryos, *pPIN1::PIN1-GFP* expression was restricted to apical cells and polarized in the plasma membrane facing the basal embryo pole (Fig. 7A). When wild-type embryos developed to the transition and heart stages, *PIN1-GFP* was expressed in the developing vasculature and cotyledon primordia (Fig. 7B, C). In *rnj* irregular globular embryos, *PIN1-GFP* was expressed asymmetrically in the outermost cell layer of the apical region (Fig. 7D). In *rnj* embryos with abnormal cotyledons, *PIN1-GFP* was distributed asymmetrically and was much stronger in one cotyledon primordium than the other (Fig. 7E).

DR5 is a synthetic auxin-responsive promoter, and a GFP-fused marker has been used to visualize the spatial pattern of auxin responses during embryogenesis (Friml *et al.*, 2003). In wild-type plants, *pDR5rev::3XVENUS-N7* expression was concentrated in the hypophysis of the globular embryos (Fig. 7F), and was also distributed in the cotyledon primordial tips and prevascular cells of embryos in the transition and heart stages (Fig. 7G, H). As well as the disruption of *PIN1* expression pattern, *pDR5::VENUS* was confusedly distributed in *rnj* embryos. We found that *pDR5rev::3XVENUS-N7* expression was restricted to the basal region of *rnj* irregular globular embryos (Fig. 7I). The *pDR5::VENUS* signal was barely detectable in the basal region of *rnj* embryos with abnormal cotyledons and was expressed asymmetrically at the tips of the initiating and developing cotyledon only (Fig. 7J, L). Therefore, auxin transport and responses associated with morphogenesis of cotyledons is disturbed in the *rnj* embryos.

In summary, we analysed the biological function of the *AtRNJ* gene in embryogenesis. *AtRNJ* is widely expressed in green tissues and reproductive organs, and its expression level is profoundly influenced by light. Homozygous *rnj* mutation leads to embryo death, which may be caused by impaired chloroplast development. In aborted embryos, cell division planes displayed abnormal orientation, and the apical-basal pattern was disturbed. Auxin transport and response was also altered in the mutant. Therefore, we surmise that *AtRNJ* may regulate embryo morphogenesis and pattern formation through controlling chloroplast development.

## Discussion

### RNJ is a plant-specific ribonuclease required for chloroplast development

In bacteria, RNJ proteins have 5′-3′ exoribonuclease activity and play important roles in rRNA maturation and 5′ stability of mRNA. Depletion of RNase J1 in *Bacillus subtilis* results in abolishment of 16S rRNA processing and cell viability (Mathy *et al.*, 2007). Three other kinds of RNJ-like metallo-beta-lactamase proteins, TRZ, CPSF73-I, and Int11, have been studied in the context of reproductive development in *Arabidopsis*. TRZ is an endonuclease that cleaves precursor molecules at the 3′-end to generate mature functional tRNA (Schiffer *et al.*, 2002). In *Arabidopsis*, four genes were found to encode tRNase Z enzymes (Supplementary Figure 1A), and AtTRZ2 was shown to be localized to chloroplasts and to be essential for embryogenesis (Canino *et al.*, 2009). CPSF73-I has endonuclease activity and catalyses the cleavage of mRNA precursors at the 3′-end in the formation of polyadenylated mRNAs (Mandel *et al.*, 2006). A single CPSF73-I protein is encoded in the genomes of *Arabidopsis*, rice, human, and yeast (Supplementary Figure 1A). Both knockdown and overexpression of *CPSF73-I* in *Arabidopsis* caused death (Xu *et al.*, 2006). Int11 is an endonuclease that is the key catalytic component that cleaves snRNA precursors (Baillat *et al.*, 2005). This protein was first studied in *Arabidopsis*, where it was designated AtCPSF73-II and was shown to be essential for early embryo development; heterozygous mutants displayed empty seed spaces as well as aborted seeds with embryos arrested at the globular stage (Xu *et al.*, 2004). Int11 homologues can also be found in human and rice, but not in yeast (Supplementary Figure 1A). Unlike these RNJ-like metallo-beta-lactamase proteins, RNJ occurs only in the nuclear genomes of plants. Further, RNJ is highly conserved in plants (Supplementary Figure 1A). In *Arabidopsis*, it had been shown that RNJ has 5′-3′ exoribonuclease and endonucleolytic activities and that it is localized in chloroplasts, plastid organelles derived from a relative of an ancestral cyanobacterium through endosymbiosis. This analysis shows that the plant *RNJ* gene may have originated from the genome of an endosymbiotic bacterium and then been transferred to the host plant genome during evolution.

A previous study concluded that a major role of RNase J is RNA surveillance to prevent the overaccumulation of antisense RNA in chloroplasts and to regulate chloroplast gene expression during post-transcriptional processes. Repression of *RNJ* expression in *Arabidopsis* and *Nicotiana benthamiana* led to chlorosis in mature leaves (Sharwood *et al.*, 2011). In our study, we found that *RNJ* is expressed broadly in green tissues and reproductive organs in *Arabidopsis* (Fig. 3) and that light induction is an important aspect of the regulation of the expression (Supplementary Figure 2). Knockout of *RNJ* caused white ovules (Fig. 1) and embryo lethality (Fig. 2). Observation of ultrastructures showed that aborted embryo cells displayed impaired chloroplast development (Fig. 4). All of the plastids in *Arabidopsis* embryos, including chloroplasts, are derived from small, non-green proplastids in meristematic cells, and the development of chloroplasts from proplastids requires the normal transcription and translation the plastid genome (Lopez-Juez and Pyke, 2005); organelle differentiation is known to be largely co-regulated by both nuclear and plastid genes (Leon *et al.*, 1998; Woodson and Chory, 2008). In *Chlamydomonas*, analysis of mutants defective in chloroplast functions led to the characterization of many nuclear-encoded proteins that regulate chloroplast gene expression (Lown *et al.*, 2001; Wostrikoff *et al.*, 2004; Raynaud *et al.*, 2007). In *Arabidopsis*, certain nuclear-encoded proteins were found to primarily control chloroplast gene expression through post-transcriptional mechanisms including transcript maturation (splicing, processing, and editing) and translation (Kleffmann *et al.*, 2004; Saha *et al.*, 2007; Sakamoto *et al.*, 2008). Therefore, AtRNJ may regulate plastid gene expression at the post-transcriptional stage, which would mean that normal *RNJ* expression is required for chloroplast development.

### Impaired chloroplasts lead to aborted embryos in *rnj* mutants

Our results show that embryo development in the *rnj* mutant is disturbed, leading to the production of irregular globular and abnormal cotyledon embryos (Fig. 2). We found that the chloroplasts in the aborted *rnj* embryos were impaired, having no stacked or well organized thylakoids (Fig. 4). It is known that plastids play versatile roles in plant growth and development, including during embryogenesis (Inaba and Ito-Inaba, 2010). During *Arabidopsis* embryogenesis, proplastids start to differentiate into chloroplasts when the embryo develops to the late globular stage (Mansfield and Briarty, 1991). Chloroplasts are an important cellular organelle that not only perform photosynthesis but are also responsible for the storage of starch and oil compounds and the synthesis of amino acids, lipids, and phytohormones (Sakamoto *et al.*, 2008). These products are involved in many metabolic and signal transduction pathways. Therefore, it is reasonable to speculate that some of these compounds are important signalling molecules that regulate embryogenesis. Interruption of proper biogenesis and development of chloroplasts in embryos has significant deleterious effects on many fundamental biological processes. Mutations of some chloroplast-localized proteins that affect important processes in chloroplasts often lead to aborted embryogenesis at early stages (Bryant *et al.*, 2011). One explanation for these observations is that the photosynthesis that occurs in chloroplasts supplies the energy for subsequent embryogenesis. On the other hand, mutations that disable the function of photosynthetic machinery usually lead to reduced pigmentation and result in white embryos with normal morphogenesis and patterning (Myouga *et al.*, 2010; Bryant *et al.*, 2011). Therefore, other metabolites such as fatty acids, amino acids, lipids, or phytohormones may participate in the signal pathways of embryogenesis. Here, we found that the *rnj* mutation impaired chloroplast development in *Arabidopsis* embryo cells, which in turn led to loss of functions important in various metabolic processes in chloroplasts. These metabolic changes may have disrupted programmed cell division and differentiation during *Arabidopsis* embryogenesis and resulted in aberrant embryo morphological development.

### RNJ is required for embryo pattern formation through regulating chloroplast function

Our results showed that *Arabidopsis rnj* embryos were of two types: irregular globular embryos and abnormal cotyledon embryos (Fig. 2). We found that defective embryo development in the mutants correlated with ectopic expression of tissue-specific genes such as *STM*, *FIL*, *ML*1, and *SCR* that are restricted, respectively, to shoot meristems, the abaxial side of cotyledon primordia, the epidermal layer, and endodermal cells in wild-type embryos (Figs 5 and 6). The irregular globular embryos did not display any such tissue specialization and then developed into an amorphous agglomeration of cells. In the irregular globular embryo, no *STM* or *FIL* signals were detected, while *ML1* was found to be expressed only in part of the outermost cells and *SCR* expression was restricted to a decreased region of endodermal cells. The other kind of *rnj* embryo had a pair of asymmetric cotyledons with a much larger angle between them than those of the wild type. The expression domains of *STM* and *FIL* were expanded in these asymmetric cotyledon embryos, yet *ML1* and *SCR* were distributed in their specific cells with abnormal division patterns. These results indicated that the *rnj* mutation led to a perturbation of apical-basal patterning in embryos. However, the expression pattern of the QC-specific gene *WOX5* was the same in both wild-type and *rnj* embryos (Fig. 6). Taken together, we surmise that AtRNJ plays an important role in the apical-basal patterning of embryos, but not for the formation of the QC in the embryonic root. Generating the apical-basal patterning is well known to be taken over by programmes that set a predictable sequence of cell divisions. Although the overall mechanism of embryo patterning remains relatively poorly understood, some functional components involved have been shown to participate in the regulation of this process (Capron *et al.*, 2009; Xiang *et al.*, 2011; Bjerkan *et al.*, 2012; Ballesteros *et al.*, 2013). Here, we suggest that AtRNJ may affect the expression patterns of certain tissue-specific genes by controlling chloroplast development.

It has been postulated that plastid development and gene expression are largely under nuclear control, a concept known as anterograde control. However, signals generated in plastids can be transduced to the nucleus and modulate nuclear gene expression. This retrograde regulation coordinates gene expression between the plastid and nuclear genomes, which is essential for maintaining plastid biogenesis and plastid function at optimal levels (Woodson and Chory, 2008). Although we know almost nothing about the nature of the retrograde signals and how they are transduced from plastids to the nucleus, potential candidate signal molecules and pathways have been proposed. These include tetrapyrrole intermediate biosynthesis, plastid gene expression (PGE), plastid redox state, and reactive oxygen species (ROS) (Pogson *et al.*, 2008; Chi *et al.*, 2013). AtRNJ is localized in the chloroplast and compensates for inefficient transcriptional termination by removal of antisense RNA, thus regulating chloroplast gene expression during post-transcriptional processes (Sharwood *et al.*, 2011). Here, we found that the expression patterns of certain nuclear genes were altered in the absence of *AtRNJ*. Therefore *AtRNJ* may participate in retrograde control and modulate nuclear gene expression by the plastid gene expression signalling pathway, and thus plays an important role in apical-basal patterning during embryogenesis.

In conclusion, the plant-specific nuclear gene *AtRNJ* encodes a chloroplast-localized ribonuclease that regulates plastid gene expression at the post-transcriptional stage and plays an essential role in chloroplast development. The *rnj* mutation leads to impaired chloroplast development in aborted embryos, which results in disrupted expression of certain nuclear genes. All of our results support the supposition that the *RNJ* gene affects embryo morphogenesis and pattern formation through controlling chloroplast development in *Arabidopsis*.

## Supplemental material

Supplementary data can be found at *JXB* online.

Supplementary Table S1. Primers used for PCR.

Supplementary Figure S1. RNase J is a metallo-beta-lactamase and conserved in plants.

Supplementary Figure S2. The functional domains of RNJ with respect to the T-DNA insertions.

Supplementary Figure S3. Embryo characteristics in wild-type, *rnj-1*/+, and *rnj-3*/+ plants.

Supplementary Figure S4. The downregulation of *RNJ* expression levels after 24h dark treatment.

## Funding

This research was supported by the National Basic Research Programme of China (2012CB944801, 2013CB126903), and the Key Grant Project of the Chinese Ministry of Education (NO. 311026).

## Supplementary Material

Supplementary Data
