# Supplementary material for: Ribonuclease J is required for chloroplast and embryo development in Arabidopsis
Source: J Exp Bot. 2015 Feb 20;66(7):2079–91. doi: 10.1093/jxb/erv010 (PMC4378637; doi:10.1093/jxb/erv010)
Supplement: Supplementary Data [file supp_66_7_2079__index.html]

Ribonuclease J is required for chloroplast and embryo development in Arabidopsis — Ribonuclease J is required for chloroplast and embryo development in Arabidopsis — Supplementary Data 

# Ribonuclease J is required for chloroplast and embryo development in *Arabidopsis*

## Supplementary Data

Data files

**Files in this Data Supplement:**

- Supplementary Data - Supplementary Data
