# Supplementary material for: Ribonuclease J is required for chloroplast and embryo development in Arabidopsis
Source: J Exp Bot. 2015 Feb 20;66(7):2079–91. doi: 10.1093/jxb/erv010 (PMC4378637; doi:10.1093/jxb/erv010)

Title: Ribonuclease J is required for chloroplast and embryo development in *Arabidopsis*

Running Title: *AtRNJ* is required for embryogenesis

Authors: Hongyu Chen, Wenxuan Zou, Jie Zhao*

Address: State Key Laboratory of Hybrid Rice, College of Life Sciences, Wuhan University, Wuhan 430072, China

***Corresponding author:** Jie Zhao

E-mail: jzhao@whu.edu.cn

Tel: 86-27-68756010

Supplemental Materials

Table S1. Primers (5' to 3') used in this study.

1.1 Primers for mutant verification		
	FP	RP
rnj-1	aaaggaaagctggcaacaaaact	gcggccaagggaagagtaatc
rnj-2	cctatactttcaccttccgcc	tgtattagggtccaatgctgg
rnj-3	tttgaatgcgtgactactccc	agattgatgaagcaccattgg
CSLB	cccatttggacgtgaatgtagacac	
1.2 Primers for complementation		
	FP	RP
RNJ-gDNA	cgcGGTACCctgggaccaagtgataccggaa	cgcGAGCTCcttgggatttggattctgcgtt
1.3 Primers for GUS/GFP fusion constructs		
	FP	RP
RNJ-Pro-GUS	gcgGTCGACctgggaccaagtgataccggaa	gcgAGATCTatcatcactgtataacaaaaccca
RNJ-Pro-GFP	gcgCTGCAGctgggaccaagtgataccggaa	gcgTCTAGAatcatcactgtataacaaaaccca
1.4 Primers for qRT-PCR		
	FP	RP
GAPDH	gagtctactggtgtcttcactg	caaggtcggacttgtattcgtg
AtRNJ-qRT	ttggtggagaggtgtttcgtg	acctcttccaattctccacg

SFig. 1 RNase J is a metallo-beta-lactamase and conserved in plants. (A) Phylogenetic tree analysis of RNJ with TRZ, CPSF73-I and CPSF73-II from *Arabidopsis* and other organisms. The tree was constructed by the Neighbor–Joining method with MEGA program 4.0. Branch numbers represent the percentage of bootstrap values in 1000 sampling replicates. Abbreviations: At, *Arabidopsis thaliana*; Hs, *Homo sapiens*; Os, *Oryza sativa*; Gm, *Glycine max*; Sb, *Sorghum bicolor*; Sc, *Saccharomyces cerevisiae*; Vv, *Vitis vinifera*; Zm, *Zea mays*. (B) Protein comparison of AtTRZs, AtCPSF73-I, AtCPSF73-II and AtRNJ showing the conserved motifs.

SFig. 2 The functional domains of RNJ with respect to the T-DNA insertions. The conserved domains of RNJ as a metallo-beta-lactamase (I-IV; A-C) are indicated along with the diagnostic amino acid residues. The Arabic number represents the specific amino acid position in the protein.

SFig. 3 The embryo characteristics in wild-type, *rnj-1/+,* and *rnj-3/*+ plants. (A-E) Embryos from the globular stage to the bent cotyledon stage in wild-type ovules. (A) embryo in globular stage; (B) embryo in transition stage; (C) embryo in heart stage; (D) embryo in torpedo stage; (E) embryo in bent cotyledon stage. (F-J) The irregular globular *rnj-1* embryos from different development stages as similar as wild-type embryos showed in (A-E). (K-O) The *rnj-1* embryos with abnormal cotyledons from different development stages as similar as wild-type embryos showed in (A-E). (P-T) The irregular globular *rnj-3* embryos from different development stages as similar as wild-type embryos showed in (A-E). (U-Y) The *rnj-3* embryos with abnormal cotyledons from different development stages as similar as wild-type embryos showed in (A-E). Scale bars = 20 μm.

SFig. 4 The down-regulation of *RNJ* expression level after 24h dark treatment. (A) Real-time quantitative PCR analysis of *RNJ* expression in 7DAG seedlings. CK, control check; Dark, 24h dark condition; DAG, day after germination. (B-C) GUS staining signals in 7DAG seedlings from *pRNJ::GUS* transgenic plants. (B) 7DAG seedling in normal light cycles; (C) 7DAG seedling with 6 days normal light cycles and 24h dark condition.

Figure S1

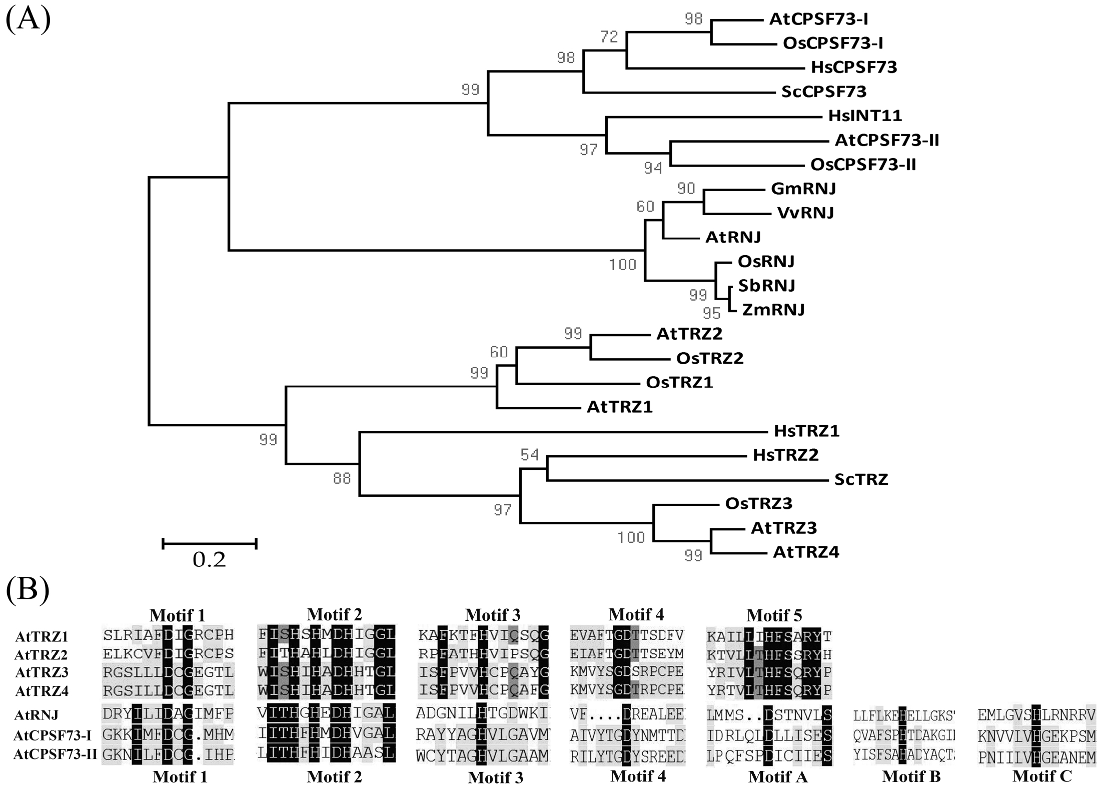

Figure S2

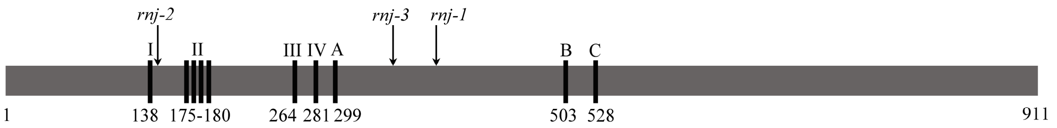

Figure S3

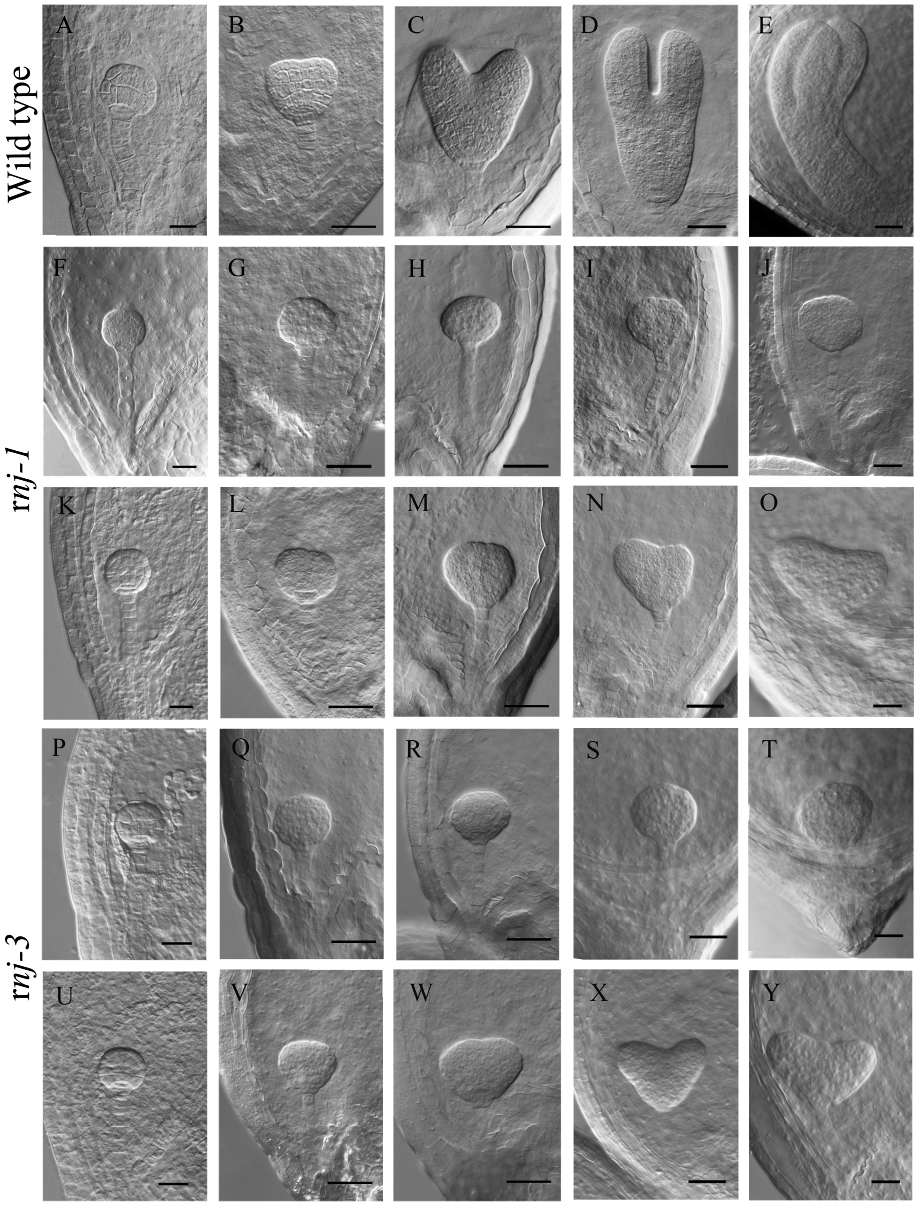

Figure S4

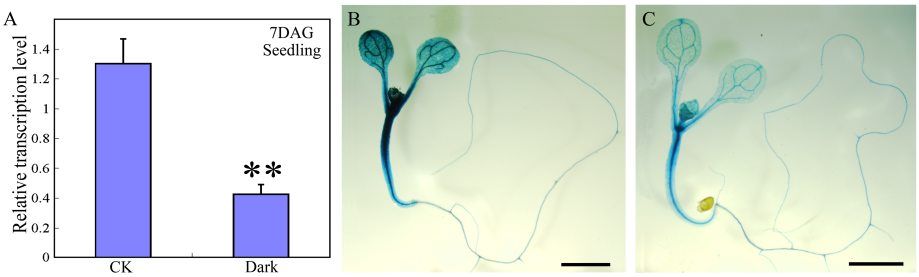

Supplement: Supplementary Data [file supp_erv010_jexbot139691_file001.pdf]
